# No evidence for transmission of SIVwrc from western red colobus monkeys (*piliocolobus badius badius*) to wild west african chimpanzees (*pan troglodytes verus*) despite high exposure through hunting

**DOI:** 10.1186/1471-2180-11-24

**Published:** 2011-02-01

**Authors:** Siv Aina J Leendertz, Sabrina Locatelli, Christophe Boesch, Claudia Kücherer, Pierre Formenty, Florian Liegeois, Ahidjo Ayouba, Martine Peeters, Fabian H Leendertz

**Affiliations:** 1 Robert Koch-Institut, Nordufer 20, 13353 Berlin, Germany; 2 Max-Planck-Institute for Evolutionary Anthropology, Deutscher Platz 6, 04103 Leipzig, Germany; 3 Norwegian School of Veterinary Science, P.O. Box 8146 dep., N-0033 Oslo, Norway; 4 Institut de Recherche pourle Developpement (IRD) and University of Montpellier 1, Montpellier, France; 5 World Health Organization, Global Alert and Response, Geneva, Switzerland

## Abstract

**Background:**

Simian Immunodeficiency Viruses (SIVs) are the precursors of Human Immunodeficiency Viruses (HIVs) which have lead to the worldwide HIV/AIDS pandemic. By studying SIVs in wild primates we can better understand the circulation of these viruses in their natural hosts and habitat, and perhaps identify factors that influence susceptibility and transmission within and between various host species. We investigated the SIV status of wild West African chimpanzees (*Pan troglodytes verus) *which frequently hunt and consume the western red colobus monkey (*Piliocolobus badius badius*), a species known to be infected to a high percentage with its specific SIV strain (SIVwrc).

**Results:**

Blood and plasma samples from 32 wild chimpanzees were tested with INNO-LIA HIV I/II Score kit to detect cross-reactive antibodies to HIV antigens. Twenty-three of the samples were also tested for antibodies to 43 specific SIV and HIV lineages, including SIVwrc. Tissue samples from all but two chimpanzees were tested for SIV by PCRs using generic SIV primers that detect all known primate lentiviruses as well as primers designed to specifically detect SIVwrc. Seventeen of the chimpanzees showed varying degrees of cross-reactivity to the HIV specific antigens in the INNO-LIA test; however no sample had antibodies to SIV or HIV strain - and lineage specific antigens in the Luminex test. No SIV DNA was found in any of the samples.

**Conclusions:**

We could not detect any conclusive trace of SIV infection from the red colobus monkeys in the chimpanzees, despite high exposure to this virus through frequent hunting. The results of our study raise interesting questions regarding the host-parasite relationship of SIVwrc and wild chimpanzees in their natural habitat.

## Background

Simian Immunodeficiency Viruses (SIVs) are the direct precursors of Human Immunodeficiency Viruses (HIVs) that have caused the HIV/AIDS pandemic in the human population [1,2]. Although the conditions and circumstances of cross-species transmission of SIVs from primates to humans remain unknown, human exposure to blood or other secretions of infected primates (chimpanzees, gorillas, sooty mangabeys) through hunting and butchering of primate bushmeat, represents the most plausible source for human infection [1-6]. Currently, serological evidence of SIV infection has been shown for more than 40 different primate species and SIV infection has been confirmed by sequence analysis in the majority of them. The routes of SIV transmission within and between host species are not fully known, however, sexual contact and biting within one species, and biting and blood-to-blood/mucosa contact (mainly observed in hunter - prey relationships) among different species provide possible infection routes for the virus [7,8]. A high genetic diversity is observed among the different SIVs, but generally each primate species is infected with a species-specific virus, which forms monophyletic lineages in phylogenetic trees. There are many examples of co-evolution between viruses and their hosts, but also cross-species transmission and recombination between distant SIVs seems not exceptional and one species can even harbour two different SIVs. The chimpanzee SIV (SIVcpz) is for example the result of cross-species transmissions as this virus is a mosaic of SIVs infecting other African primates. The genome of the virus consists partly of nucleic acid sequences from red capped mangabey SIV (SIVrcm), and partly of sequences from the ancestor of SIVs infecting greater spot-nosed (SIVgsn), mona (SIVmon) or mustached monkey (SIVmus) [9-11]. Chimpanzees are known to hunt monkeys for food, and most probably, the recombination of these monkey viruses occurred within chimpanzees and gave rise to the common ancestor of today's SIVcpz lineages, which were subsequently transmitted to gorillas [5].

Despite the increasing number of SIV lineages that have been described recently, our knowledge on SIV in their natural hosts still remains limited. This is because only few viruses have been characterized for each species and there is a major bias in geographical sampling. By studying SIVs in wild primates in their natural habitat we can better understand the circulation and transmission of these viruses within and between different primate species and perhaps identify factors that play a role in viral adaptation to new hosts among different primate species [12-14].

Of the four chimpanzee subspecies, only *Pan troglodytes troglodytes *and *Pan troglodytes schweinfurthii *in Central/East Africa have been shown to harbour SIVcpz [1,11,15-18]. The two West African chimpanzee subspecies, *Pan troglodytes ellioti *and *Pan troglodytes verus*, appear to be free from SIVcpz infection. Therefore it is hypothesized that this virus was introduced after the evolutionary divergence and geographical separation of the West African subspecies from the Central/East subspecies [11,15]. To test for SIVcpz in *P. t. verus*, more than 1500 captive chimpanzees of this subspecies have been screened for this virus. However, these chimpanzees do not represent the wild population since only 447 were wild-born and have mainly been captured as infants, when they are less likely to be infected [15,19]. Therefore, it remains important to continue to collect data on wild living chimpanzees from this subspecies. To date, the only study on wild living *P. t. verus *has been based on 28 faecal samples from a population in Taï National Park, Côte d'Ivoire [16].

The chimpanzees of Taï National Park have been under human observation for more than 30 years [20] and are known to hunt and consume monkeys frequently. When hunting, the chimpanzees bite their prey and are sometimes bitten in return. The prey is consumed almost entirely, which means that many bones are crushed which could cause lesions in the oral cavity and result in direct blood to blood contact. They hunt weekly throughout the year and usually every day in the hunting season from September to November, and 80% of their prey consist of western red colobus monkeys (*Piliocolobus badius badius*) [20]. These red colobus monkeys harbour high levels of their own species specific strain of SIV (SIVwrc) as well as two other retroviruses; Simian T-cell Lymphotrophic Virus type 1 (STLV-1wrc) and Simian Foamy Virus (SFVwrc) [21-25].

Based on the SIVwrc prevalence data from this red colobus population (50 to 82% of the population is positive [21]) and based on hunting data from the Taї Chimpanzee Project [20], we estimate that adult male chimpanzees are yearly exposed to approximately 45 kilograms of SIV infected red colobus tissue. Therefore the chimpanzees are exposed to high levels of SIVwrc through biting, blood-to-blood/mucosa contact and ingestion of their prey. This may provide possible infection routes for the virus, although the modes of SIV transmission are not fully known [7,8]. It has already been documented that the other two retroviruses harboured by the red colobus monkeys in Taї National Park; STLV-1wrc and SFVwrc, are transmitted to the Taї chimpanzee population (individuals are included in the present study) most likely through hunting and meat consumption [22,23]. Further, in chimpanzee subspecies where the chimpanzee lentivirus, SIVcpz, has been documented, it is believed that this mosaic virus was initially acquired through hunting and consumption of infected monkey prey species [9-11]. Such evidence of retroviral transmissions through hunting and consumption of primates makes it possible that also SIVwrc from red colobus monkeys can be transmitted to the *P. t. verus *chimpanzees. The aim of this study was therefore to investigate the SIV status of these wild chimpanzees, and to determine if transmission of SIVwrc occurs from red colobus monkeys to the chimpanzees through their natural and frequent hunting and meat-eating behaviour.

## Results

### Chimpanzee samples

Table 1 summarises the characteristics of the chimpanzees analyzed in this study; name, social group, sex, age, cause of death or sampling, and samples available for antibody testing and PCRs. Twenty chimpanzees were older than ten years (subadults/adults) (9 males and 11 females). The remaining 12 chimpanzees were younger (juveniles/infants) (8 males and 4 females). Chimpanzees over ten years start to participate in hunting but rarely have access to the meat until they reach adulthood (15 years), while younger chimpanzees have access to meat through their mothers [20]. Fourteen of the chimpanzees died of respiratory disease, six died of anthrax, two died of leopard attack, one died of an intergroup fight, one died of heart failure, and four died of yet unknown causes [26-28; C. Boesch and F. Leendertz, unpublished data].

**Table 1:** Summary of chimpanzees, samples, and results for INNO-LIA test, Luminex test and PCRs.

Name	Group	Sex	Age at testing	Cause of death/ sampling	Sample for INNOLIA Luminex	INNOLIA reactivity	Luminex results	Sample for PCR	PCR
Kady	Middle	F	Adult	Heart failure	Blood	gp41 (+/-)	Neg	Spleen	Neg

Leo	Middle	M	Adult	Anthrax	Blood	spg120 (1+) gp41 (1+)	Neg	Spleen	Neg

Dorry	North	F	10 years	Anthrax	Blood	gp41 (1+)	Not done	Liver	Neg

Loukoum	North	F	Adult	Respiratory disease	Blood	gp41 (+/-)	Neg	Liver	Neg^4)^

Leonardo	North	M	2 years	Respiratory disease	Blood	-	Neg	Spleen	Neg^4)^

Lefkas	North	M	8 years	Respiratory disease	Blood	-	Neg	Liver	Neg^4)^

Rafiki	South	M	Adult	Leopard attack	Blood	-	Not done	Spleen	Neg

Noah	Middle	M	7 years	Anthrax	Blood	-	Not done	Spleen	Neg

Tita	South	F	Adult	Leopard attack	Blood	-	Neg	Spleen	Neg^4)^

Olduvai	South	M	8 years	Anthrax	Blood	spg120 (1+) gp41 (+/-) spg 105 (1+)	Neg	Spleen	Neg

Gargantua	North	M	10 years	Anthrax	Blood	-	Not done	Muscle^1)^	Neg

Gisele	North	F	5 years	Anthrax	Blood	-	Not done	Muscle^1)^	Neg

Virunga	South	F	Adult	Respiratory disease	Blood	-	Neg	Spleen	Neg

Ophelia	South	F	<1 year	Respiratory disease	Blood	-	Neg	Spleen	Neg

Orest	South	M	5 years	Respiratory disease	Blood	-	Neg	Spleen	Neg

Nerone	East	M	Adult	Intergroup fight	Blood	-	Not done	Heart, lung^2)^	Neg

Candy	East	F	Adult	Respiratory disease	Blood	-	Neg	Spleen	Neg

Ishas baby	South	M	<1 year	Respiratory disease	Blood	-	Neg	Spleen	Neg

Vasco	East	M	Adult	Respiratory disease	Blood	-	Neg	Spleen	Neg

Porthos	East	M	Adult	Under investigation	Blood	-	neg	Lymphnode	Neg

Dartagnan	East	M	Adult	Under investigation	Blood	-	Neg	Spleen	Neg

Iome	East	F	<2 years	Unknown	Blood	-	Neg	Spleen	Neg

Kahula	East	M	4 years	Under investigation	Blood	-	Neg	Spleen	Neg

Sagu	South	M	Adult	Surgical intervention	Serum	-	Neg	Buffy coat	Neg

Zora	South	F	Adult	Sample collected from environment	Blood	-	Not done	Spleen^3)^	Neg

Coco	South	F	Adult	Sample collected from environment	Blood	-	Not done	No material available	Not done

Atra	South	F	Adult	Respiratory disease	Blood	-	Neg	Spleen	Neg

Stranger	East	M	Adult	Sample collected from environment	Blood	-	Not done	No material available	Not done

Olivia	South	F	Adult	Respiratory disease	Blood	-	Neg	Spleen	Neg

Louise	South	F	Adult	Respiratory disease	Blood	-	Neg	Spleen	Neg

Akruba	South	F	5 years	Respiratory disease	Blood	-	Neg	Lung	Neg

Akwaba	South	M	1 year	Respiratory disease	Blood	-	Neg	Spleen	Neg

Adult chimpanzees are older than 15 years. For the INNO-LIA test result, only reactivity equal to, or stronger than, control cut off band (+/-) is included in the table, others are marked as "-". PCR testing was performed using SIVwrc specific primers as well as SIV generic primers (DR1/DR2) except for: ^1) ^Sample tested with SIVwrc PCR only, ^2) ^Heart tested with SIVwrc PCR, lung tested with SIVgeneric PCR, ^3) ^Sample became available late in the study, tested with SIVgeneric PCR only, ^4) ^Samples additionally tested by further PCRs as shown in Table 2.

### Detection of antibodies to HIV/SIV

**Figure 1:**
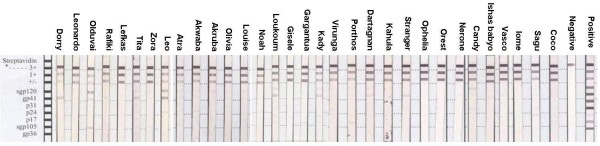
**Results from the INNO-LIA test**. Names are according names of chimpanzees sampled; Positive = positive control serum; Negative = negative control serum.

Samples from 17 of the 32 chimpanzees showed weak but visible reactions to HIV-1/HIV-2 antigens in the INNO-LIA HIV score test (Table 1; Figure 1). The strongest reactions were seen for Leo, who scored equal or stronger than 1+ for reactions to HIV-1 env proteins (spg120 and gp41) in addition to a weak reaction to p24, and Olduvai had cross-reactivity with HIV-1 and HIV-2 antigens; equal to 1+ to spg120 and spg105 and equal to cut-off control for gp41. Dorry scored equal to 1+ to gp41. Loukoum and Kady both had reactions equal to cut-off control for gp41 and a faint reaction to spg120. Kady had in addition a faint reaction to p17. Twelve other chimpanzees (Sagu, Rafiki, Porthos, Dartagnan, Candy, Vasco, Louise, Tita, Stranger, Gargantua, Akruba and Akwaba) had faint reactions, below cut-off, to one or two of the following antigens: sgp120, gp41, p24, p17 or sgp105. Considering all visible reactions together, there were more frequent reactions in the adults/subadults than in the juvenile/infants (14/20 [70%] and 3/12 [25%], respectively) and more frequent reactions to HIV-1 specific antibodies than to HIV-2 specific antibodies (reactions in 13 and three chimpanzees, respectively).

Using the Luminex test, each of the 23 samples were negative against all the peptides representing the different SIV lineages, including SIV lineages described for the primate species living in the region (SIVwrc, SIVolc, SIVsmm, and SIVgsn, although SIV infection has not yet been documented for this latter species in Taï) and all known great ape SIVs (SIVcpz*Ptt *SIVcpz*Pts*, SIVgor) and HIVs (HIV-1 group M, N and O and HIV-2) with a signal to cut-off ratio below 1, in contrast to human, monkey and chimpanzees positive controls.

### PCR analyses

None of the samples from the chimpanzees were positive for any SIV strain; neither when using the generic SIV PCR or the SIVwrc-specific PCR in *pol*. Also the additional PCRs with SIVwrc specific primers amplifying *pol*, *env *and *gag *fragments of SIVwrc/SIVolc/SIVcol sequences and primers amplifying *gag *and *env *regions of SIVsmm were negative. The quality of all PCRs was confirmed with positive control samples known to be infected with the respective viruses.

## Discussion

There are a number of interesting questions regarding the transmission and natural history of SIV infections in wild chimpanzees; an infection which entered into and adapted to the human population and caused the global AIDS pandemic [2]. It is presumed that the chimpanzees first acquired the infection through hunting and consumption of monkey prey infected each with their own species specific strains of SIV, which at some point in time recombined and persisted in the chimpanzee host [9-11]. To date, only this recombinant strain of SIV, known as SIVcpz, has been detected in wild chimpanzees [29] and one question that arises is: How easily are individual SIV strains from monkeys transmitted to chimpanzee populations, irrespective of subspecies, and do such infections persist?

We investigated this question through studying the natural hunter-prey relationship between wild chimpanzees (*P. t. verus*) and highly SIV-infected red colobus monkeys (*P. b. badius*) in the tropical rainforest of Taï National Park in Côte d'Ivoire, West Africa [21,30]. Eight other diurnal monkey species live in this forest, including olive colobus monkeys (*Procolobus verus*), great spot-nosed monkeys (*Cercopithecus nictitans*) and sooty mangabeys (*Cercocebus atys*) which are also known to harbour species-specific SIVs: SIVolc, SIVgsn and SIVsmm, respectively [4,24,31]. However, according to more than 30 years of behavioural observations, red colobus is the preferred prey of the chimpanzees, whereas capture of greater spot-nosed monkeys has not been observed and olive colobus and sooty mangabeys are hunted extremely rarely. For example, over a twelve year period, the chimpanzees were seen to capture only six olive colobus and one sooty mangabey, while red colobus monkeys were captured 215 times [20]. Therefore, the exposure to these respective SIV strains through hunting is very low in comparison to the exposure to the SIVwrc strain carried by the red colobus monkeys, which the chimpanzees are frequently in close contact with. In addition, the prevalence of SIV in this monkey species in Taï National Park is among one of the highest documented in wild primates to date. Western red colobus represent a substantial reservoir to which chimpanzees, as well as human bushmeat hunters, are exposed [21]. As an example, we calculated that Sagu, an adult male in our study who was approximately 20 years old when he died, could have eaten nearly 250 kilograms of SIVwrc infected red colobus tissue in his life.

Despite the enormous infection pressure, we could not detect SIVwrc (or any other strain of SIV) in blood and tissue samples from the chimpanzees. Theoretically, these chimpanzees could carry a SIV strain which is not detectable by the PCR methods used in this study. Alternatively, the level of SIVwrc viraemia is so low that it can not be detected by the PCR methods used. This could be in particular true for the 2 chimpanzees for which only samples of muscle were available. However, as no SIV-specific antibodies were detected with the Luminex test it is more plausible that no persistent SIV infection exists in these chimpanzees, although about half of the chimpanzees showed some cross-reactions to the HIV-antigens on the INNO-LIA HIVI/II Score kit. The strongest reactions were observed in samples from Leo and Olduvai, and their test results were HIV positive, according to the test manufacturer's criteria (two or more bands stronger than the minimum control band [the +/- band]). For another chimpanzee, Dorry, the result was indeterminate (one band stronger than the minimum control band). For other chimpanzees where weak reactions were seen, the results are considered negative for this HIV-test. It could be that there is a difference in sensitivity and specificity of HIV antibody detection of the Luminex and INNO-LIA tests, but it is also likely that the reactivity to HIV antigens in the INNO-LIA test was due to false positive cross-reaction phenomena due to other causes than HIV/SIV infection, such as observed in human HIV testing, especially in Africa [32]. It has been shown that the INNO-LIA test produces false positive results also in other primate species. In *C. nictitans *and *C. cephus *the estimated prevalence based on INNO-LIA results is higher than that estimated using lineage specific antigens, and samples from *C. pogonias, L. albigena *and *C. agilis*, that were cross-reacting with some HIV antigens on the INNO- LIA test, were negative with SIV lineage ELISAs and PCR [33,34]. Therefore the reactivity we observed with the INNO-LIA testing of the chimpanzee samples is most likely a false positive non-specific cross-reactivity, as no specific antibody reaction to SIVwrc, or any other known SIV and HIV strain, could be detected by SIV/HIV lineage specific Luminex EIAs.

It was not surprising that the *P. t. verus *chimpanzees were negative for SIVcpz, as this virus is believed to have been introduced into two other, Central/East chimpanzee subspecies (*P. t. troglodytes *and *P. t. schweinfurthii*) after the evolutionary split from the Western chimpanzee subspecies [15]. It was however interesting that we could not detect any SIVwrc infection, considering the high exposure of this virus.

Our results show that there was no detectable specific antibody reaction to, or infection with, SIV from red colobus monkeys (or any other primates), despite high and frequent exposure to the virus. This seemingly resistance of the chimpanzees to SIVwrc could be due to immunological factors or mechanisms, or lack of these, which are important for the recognition and subsequent establishment or rejection of immunodeficiency viruses [35-38]. HIV research is much focused on these mechanisms, especially in certain individuals that remain persistently seronegative despite known exposure to HIV [39]. *P. t. verus *chimpanzees are however not totally resistant to immunodeficiency virus infections in general, as susceptibility of captive chimpanzees of this subspecies to HIV, SIV, and co-infections of the two viruses, has been documented [7]. In wild chimpanzees (*P. t. troglodytes *and *P. t. schweinfurthii*) no other SIV strain than the chimpanzee specific SIVcpz has been detected to date [1,5,18], which suggests that the chimpanzees' susceptibility to individual SIV strains from monkeys is low. SIVcpz is a mosaic consisting partly of SIV from red capped mangabey and partly of one of the SIV strains in greater spot-nosed monkey, mona monkey or mustached monkey [9,10]. Only one of these species, the greater spot-nosed monkey (*C. nictitants*), lives in the Taï forest. These monkeys are however rare in this forest, the chimpanzees have never been observed to hunt them, and there is also no evidence yet that they are SIV infected, although only few animals have been tested [20,31]. Interestingly and comparably to what we report about the chimpanzees, no SIVwrc infections have so far been documented in humans, who also frequently hunt red colobus monkeys [40]. We could also speculate whether the SIV status of the chimpanzees in the Taï National Park would be different had they hunted sooty mangabeys more frequently. The sooty mangabey population from this national park harbours the sooty mangabey strain of SIV (SIVsmm) which crossed the species barrier at least 8 times and infected humans through bushmeat hunting, and then became HIV-2 [4]. The genetic and physiologic similarities between humans and chimpanzees and also the similar susceptibility to specific infections, suggest that such transmission could also occur from sooty mangabeys to chimpanzees, if an efficient transmission pathway existed.

## Conclusion

We could not detect any conclusive sign of infection with SIVwrc in the *P. t. verus *chimpanzees in Taï National Park, despite exposure of highly infected red colobus. However, the frequent hunting and consumption of red colobus by the chimpanzees represents a transmission pathway for other simian retroviruses between these two host species. It remains to be determined which factors that seemingly protect these chimpanzees from infection, and whether the local human population, frequently exposed to meat and organs of the red colobus in this region, is free of SIVwrc infections. This knowledge on natural SIV infections in wild primate populations might benefit human HIV/AIDS research [13,29].

## Methods

### Samples

Wild chimpanzees (*P. t. verus*) in the tropical rainforest of Taï National Park (5°15'-6°07'N, 7°25'-7°54'W), Côte d'Ivoire, have been studied for behavioural research for more than 30 years [20]. As part of the project's veterinary monitoring, blood, muscle and samples from internal organs of 28 chimpanzee carcasses were collected over the last 12 years [26]. Previous research has shown that SIV can be detected in these types of tissues [21]. Table 1 summarises the chimpanzees name, social group, sex, age, cause of death or sampling, and samples available for antibody testing and PCRs. Samples from 3 chimpanzees bleeding after a violent encounter with other chimpanzees were collected from the environment and from 1 chimpanzee plasma was collected during surgical intervention [26-28; F. Leendertz, unpublished data]. Whole blood was collected from dead chimpanzees or from the environment from 31 chimpanzees; for one chimpanzee serum from fresh blood was obtained. The samples were transported on ice to the forest camp and frozen in liquid nitrogen. The samples were transported on dry ice to the Robert Koch Institute, Berlin, and stored in -80°C until analyses. The work was performed under the permission of the according authorities from Côte d'Ivoire.

### HIV antibody testing

We tested samples from 32 chimpanzees with the INNO-LIA HIV I/II Score kit (Innogenetics, Gent, Belgium). The test is a line immuno-assay which is a commonly accepted and widely used confirmatory test for HIV [32]. This test has also been commonly used to detect HIV cross-reactive antibodies in non-human primates and identified a large number of new SIV lineages, but positive samples in non-human primates should be confirmed with other more specific antibody tests and/or PCRs as false positive reactions can occur [33,34,41,42]. The test is designed for use on serum or plasma samples. We dissolved whole blood, which was preserved frozen since collection, with 0.2 ml of PBS and used the supernatant for the test, as well as plasma from one chimpanzee (blood centrifuged directly after collection under anaesthesia). In the INNO-LIA HIV I/II Score kit antigens from HIV-1 and HIV-2 are coated as discrete lines on a nylon strip. There are five HIV-1 antigens: sgp120 and gp41, which detect specific antibodies to the HIV-1 envelope, and p31, p24, and p17, which detect antibodies to HIV-1 *pol *and *gag *but may also cross react with HIV-2. The antigens gp36 and sgp105 are applied to detect antibodies to HIV-2 envelope proteins. For each antigen a coloured band develop in proportion to the HIV-antibodies present in the sample. The strength of the reaction is read in comparison to control bands on each strip; one for +/- cut off level, one for 1+ reaction and one for 3+ reaction. Two samples (Leo and Olduvai) were retested in another batch to confirm the results.

### Specific SIV and HIV antibody testing

Out of the 32 chimpanzees, sufficient material for further antibody testing was available from 23 chimpanzees. These samples were tested for antibodies against a panel of 43 antigens consisting of 18 peptides of the gp41 immunodominant region representing the majority of all known HIV/SIV lineages, including SIVwrc, and 25 peptides of the V3 region, including the four groups of HIV-1, HIV-2, SIVcol and representatives of the different SIVcpz/gor lineages that circulate among chimpanzees and gorillas in central Africa [17,33]. To that end, we used a newly developed assay based on the Luminex technology. This new Luminex test is comparable to SIV specific ELISAs [[33,43], Ayouba et al., manuscript in preparation]. This test is also an EIA, with the reaction support consisting of calibrated polystyrene beads on which peptides are covalently bound. Each peptide was immobilized on a distinct bead set with a unique fluorescence wavelength. Once covalently linked to bead, the 43 different peptides were mixed and distributed in wells of a semi-permeable ELISA plate like support. Diluted (100 μl, 1/200) antibodies-containing fluids (serum, plasma or whole blood) were then added and incubated at room temperature for an hour with continuous shaking. After washing, 50 μl of a biotin-labelled anti-human IgG was added in each well and the plate was incubated 30 minutes at room temperature, while shaking. After a second series of washing, R-phycoerythrine-labelled streptavidine was added for 10 minutes and washed out afterwards. The complex consisting of beads-peptide and the different additives was resuspended in buffer and read on a BioPlex-200 (BioRad, Marnes la Coquette, France). With the Luminex technology, each bead set is sorted in a specific area of a 2 dimensional display, according to its wavelength of fluorescence, like in flow cytometry. For each sample and for each antigen, results are expressed as median fluorescence intensity per 100 beads. Cut-off values have been calculated for each of the 43 peptides from their reactivities against 95 SIV negative non-human primates' samples and 50 HIV negative human plasma. Samples presenting MFI higher than 500 against a given were considered positive for that peptide.

### PCR analyses

For PCRs the following tissues were used: spleen (n = 21), liver (n = 3), muscle (n = 2), heart and/or lung (n = 2), lymphnode (n = 1) and buffy-coat (n = 1). For 2 chimpanzees no material was available for PCR analyses.

DNA was extracted with the DNA tissue (or blood) kit (Qiagen, Hilden, Germany). Samples were tested with a generic SIV PCR known to detect most primate Lentiviruses [44]. We used the primers DR1 (TRC AYA CAG GRG CWG AYG A) and DR2 (AIA DRT CAT CCA TRT AYT G) in the first round PCR and primers DR4 (GGI ATW CCI CAY CCD GCA GG) and DR5 (GGI GAY CCY TTC CAY CCY TGH GG) in a nested PCR. The cycling conditions were 94°C for 2 minutes, 30 × [94°C for 15 seconds, 50°C decreasing by 0.5°C each cycle to 35°C for 30 seconds, 72°C for 1 minute], 15 × [94°C for 15 seconds, 50°C for 30 seconds, 72°C for 1 minute], 72°C for 5 minutes, then cooling to 4°C The expected amplicon size was 194 bp. Samples were also tested specifically for SIVwrc with a semi-nested PCR with primers specifically designed for the detection of *pol *regions of SIVs from the western red colobus/olive colobus lineage (SIVwrc S1 [CATGGCAAATGGATTGTACTCA], SIVwrc R2 [GTGCCATTGCTAATGCTGTTTC], SIVwrc S3 [CCAAATTCTTGTTCT ATCCCTAACC], and SIVwrc R3 [AGCAAAAATCATATCAGCAGAAGAT]). These primers were based on SIVwrc and SIVolc sequences published by Courgnaud and colleagues [24]. We used SIVwrc S1 and SIVwrc R2 in the first round PCR, and SIVwrc S1 and SIVwrc R3 (expected amplicon size approximately 250 bp), and SIVwrc S3 and SIVwrc R2 (expected amplicon size approximately 300 bp) in two parallel semi-nested PCRs. The cycler conditions were 94°C for 5 minutes, 30 × [94°C for 15 seconds, 55°C for 30 seconds, 72°C for 30 seconds], 72°C for 10 minutes, then cooling to 4°C. The PCRs included positive control samples from confirmed SIVwrc positive red colobus monkeys [21]. PCR products were visualised with gel electrophoresis. A subset of samples (n = 4; Loukoum, Leonardo, Lefkas, Tita) was also tested with additional primers targeting SIVwrc/SIVolc/SIVcol in the *gag, env *and *pol *regions and primers amplifying *gag *and *env *regions of SIVsmm isolated from sooty mangabeys (Table 2).

**Table 2:** Additional PCRs for SIV testing of a subset of samples (n = 4).

Primers tested	Primer sequences	Estimated amplicon size	Region targeted	Reference
DR1-DR2/DR4-DR5	DR1 (5'-TRCAYACAGGRGCWGAYGA-3')	800	*Pol*	[44]
	DR2 (5'-AIADRTCATCCATRTAYTG -3')			
	DR4 (5'-GGIATWCCICAYCCDGCAGG-3')	200		
	DR5 (5'-GGIGAYCCYTTCCAYCCYTGHGG -3')			
	
polOR-polis4/polis2uni2	polOR(5'-ACBACYGCNCCTTCHCCTTTC -3')	800	*Pol*	[10]
	polis4(5'-CCAGCNCACAAAGGNATAGGAGG-3')			
	polis2(5'-TGGCARATRGAYTGYACNCAYNTRGAA-3')	650		
	uni2(5'-CCCCTATTCCTCCCCTTCTTTTAAAA -3')			

wrcpol	wrcpolF1 (5'-TAGGGACAGAAAGTATAGTAATHTGG-3')	1100	*Pol*	[25]
	wrcpolR1 (5'-GCCATWGCYAA TGCTGTTTC-3')			
	wrcpolF2 (5'AGAGACAGTAAGGAAGGGAAAGCAGG-3')	650		
	wrcpolR2 (5'-GTTCWATTCCTAACCACCAGCADA-3')			

wrcenv	wrcenvF1 (5'-TGGC AGTGGGACAAAAATATAAAC-3')	750	*Env*	[25]
	wrcenvR1 (5'-CTGGCAGTCCCTCTTCCA AGTT GT-3')			
	wrcenvF2 (5'TGATAGGGMTGGCTCCTGGTGATG3')	550		
	wrcenvR2 (5'-AATCCCCATTTYAACCAGTTCCA-3')			

wrcgag	wrcgagF1 (5'-ATDGAGGATAGAGGNTTTGGAGC-3')	600	*Gag*	[46]
	wrcgagR1 (5'-GCCCTCCTACTCCTTGACATGC-3')			
	wrcgagF2 (5'-CCAACAGGGTCAGATATAGCAG-3')	250		
	wrcgagR2 (5'-ACTTCTGGGGCTCCTTGTTCTGCTC-3')			

olcpol	olcpolF1(5-TAGATACAGGRGCAGATGAYACAGTAAT-3')	700	*Pol*	S. Locatelli, unpublished data
	olcpolR1 (5'TCCAYCCYTGAGGHARYACATTATA-3')			
	olcpolF2 (5'-CTAGAATWATWGGRGGRATAGGRGG-3')	300		
	olcpolR2 (5'-ATYTTWCCTTCTKCTTCYARTCTRTCACA-3')			

bwcpol	bwcpolF1 (5'-TAGATACAGGAGCAGATGATACAGT-3')	1000	*pol*	S. Locatelli, unpublished data
	bwcpolR1 (5'-ATTDCCYCCTATCCCTTTATGWGC-3')			
	bwcpolF2 (5'-AGAYTRGAAGCAGARGGAAAAAT-3')	600		
	bwcpolR2 (5'-TCCYACCAATTTYTGTAYATCATTTACTGT-3')			

polis4-SIVenvR/lhoenv	polis4(5'-CCAGCNCACAAAGGNATAGGAGG-3')	3600	*Env*	[47]
	SIVENVR (5'-YTBYTGCTGCTGCAMTATCCC-3')			
	lhoenvF2 (5'- AATCAGATAGTNYAGCAAGCATGG-3')	600		
	lhoenvR2 (5'-CCATTAAAKCCAAAGAAGCTACT-3')			

polis4/PolOr polF2/polR2	polOR(5'-ACBACYGCNCCTTCHCCTTTC -3')	800	*Pol*	[48]
	polis4(5'-CCAGCNCACAAAGGNATAGGAGG-3')			
	POLF2 (GGAAGTGGATACTTAGAAGCAGAAGT-3')	330		[5]
	POLR2 (5'-CCCAATCCCCCCTTTTCTTTTAAAATT-3')			
gp40F1-gp41R1 gp46F2/gp48R2	gp40F1(5'-TCTTAGGAGCAGCAGGAAGCACTATGGG-3')	850	*Env*	[49]
	gp41R1(5'- AACGACAAAGGTGAGTATCCCTGCCTAA-3')			
	gp46F2(5'-ACAATTATTGTCTGGTATAGTGCAACAGCA-3')	445		
	gp48R2(5'-TCCTACTATCATTATGAATATTTTTATATA-3')			
SPBS-wrcgagR1/SPBS-wrcgagR2	SPBS (5'-GGCGCCCGAACAGGGACTTG-3')	1500	LTR-*gag*	M. Peeters, unpublished data

smmgag	smmgagF1 (5'-TGGGAGATGGGCGCGAGAAACTCCGTC-3')	1000	*gag*	[50]
	smmgagR1 (5'-ATCAGCAGTGTCTGTGTCATCCAATT-3')			
	smmgagF2 (5'-AGGGAAAAAAGCAGATGAATTAGAA--3')	800		
	smmgagR2 (5'-GCTCTTGTAGAAYCTATCTACATA-3')			

smmenv (gp41)	smmenvF1 (5'-GCTACGGCAGGTTCTGCAATGGG-3')	650	*env*	[50]
	smmenvR1 (5'-CTGGTCCTTGCGGATATGGATCTG-3')			
	smmenvF2 (5'-GCTGTCCGCTCAGTCCCGGACTTT-3')	490		
	smmenvR2 (5'-GGAGGAGAACACTGGCCTATA-3')			

Y = C/T, W = A/T, R = A/G, H = A/C/T, B = C/G/T, S = G/C, K = G/T, D = A/G/T, N = A/C/T/G Y = C/T, M = A/C, K = G

Sequencing of any suspicious bands that appeared on subsequent gel electrophoresis was performed in both directions using the Sanger method, with all PCR products being sequenced on both strands. Sequences were compared to the public database using NCBI BLAST [45].

## Authors' contributions

SAJL, PF and FHL collected samples. SAJL, SL, CK, FL, AA, MP and FHL performed or supervised the laboratory analyses. SAJL, CB, MP and FHL designed the study and wrote the manuscript with contributions from all authors. All authors approved the final manuscript.
